# Critical metrology of minimally accessible anisotropic spin chains

**DOI:** 10.1038/s41598-024-70307-8

**Published:** 2024-08-27

**Authors:** Marco Adani, Simone Cavazzoni, Berihu Teklu, Paolo Bordone, Matteo G. A. Paris

**Affiliations:** 1 Center for Cyber-Physical Systems (C2PS), Khalifa University, Abu Dhabi, 127788 United Arab Emirates; 2 Dipartimento di Scienze Fisiche, Informatiche e Matematiche, Università di Modena e Reggio Emilia, 41125 Modena, Italy; 3 Department of Mathematics, College of Computing and Mathematical Sciences, Khalifa University, 127788 Abu Dhabi, United Arab Emirates; 4 Centro S3, CNR-Istituto di Nanoscienze, 41125 Modena, Italy; 5 Quantum Technology Lab, Università degli Studi di Milano, 20133 Milan, Italy

**Keywords:** Quantum Fisher information, Spin chain, Phase transition, Criticality, Information theory and computation, Quantum physics

## Abstract

We address quantum metrology in critical spin chains with anisotropy and Dzyaloshinskii–Moriya (DM) interaction, and show how local and quasi-local measurements may be exploited to characterize global properties of the systems. In particular, we evaluate the classical (magnetization) and quantum Fisher information of the relevant parameters for the density matrix of a single spin and that of a pair of spins ranging from nearest to sixth-nearest neighbors, to the limiting case of very distant spins. Our results allow us to elucidate the role of the different parameters and to individuate the optimal working regimes for the precise characterization of the system, also clarifying the effects of correlations on the estimation precision.

## Introduction

Quantum phase transitions (QPTs) in many-body systems occur at zero temperature driven by variations of the couplings and/or of external parameters^1^. The relationship between both classical and quantum phase transitions (PTs) and estimation theory is profound and indissoluble. Near (Q)PTs,(quantum) states may be discriminated with a high accuracy, because of the enhanced Quantum Fisher Information (QFI)^2^. Recently, researchers have addressed phase transitions for quantum sensing^3–8^ and experimentally implemented in trapped ions^9^ and Rydberg atoms^10^. These collective phenomena enable the development and model of extremely precise detectors and sensors^11–13^. Within this framework, the Heisenberg XY model with anisotropy and Dzyaloshinskii–Moriya (DM) interaction describes a wide range of physical systems, exhibiting multiple phase transitions, making it an excellent candidate for testing metrological protocols and schemes for detecting quantum phase transitions both with global or (quasi)-local measurements. The anisotropy and DM interaction terms describe a wide range of physical systems, from relatively simple Hamiltonians, such as the Ising model, to more complex systems incorporating inhomogeneous interactions and spin-orbit coupling^14–23^. This model has been computationally investigated^24–29^, and found applications in studying materials of technological interest^30–32^, experimental implementations^33^, and in describing surface and interface phenomena^34–37^. Moreover, DM anisotropy recently found also application in quantum information and technology^38–44^. Our analysis extends the application of the model to quantum metrology, and paves the way to realize precise extended sensors requiring only (quasi-)local readout schemes.

In this paper, we demonstrate that local or quasi-local measurements, performed on only one or two spins, may be exploited to detect quantum phase discrimination and to precisely characterize the system^45–47^. Unlike the previous literature, we introduce the single spin reduced density matrix, exploiting its metrological characteristics. For the two-spin reduced density matrix, we are going beyond the nearest neighbors spins^48^ to characterize the effect of correlations on quantum phase transitions.

More specifically, we evaluate the classical (magnetization) and quantum Fisher information matrices of the relevant parameters of the density matrix of a single spin and that of a pair of spins. Additionally, we investigate how quantum correlations influence the optimal distance between the two spins, with the goal of maximizing the QFI and thereby optimizing the estimation of the Hamiltonian parameters.

The manuscript is structured as follows: In “Model and methods”, we introduce the physical model and the theoretical methodologies adopted to investigate the phases for local and quasi-local measurements. In “Quantum and classical Fisher information theory”, we introduce the basics of information theory and the quantities monitored to theoretically and practically discriminate the phases of the physical model. Sections “Local measurements” and “Quasi local measurements and the role of correlations” present the results of the analysis, distinguishing between measurements of local properties of the system and measurements that involve correlations among the elements of the system. Finally, in “Conclusions”, we summarize the main results and highlight differences between the methodologies adopted. In Appendix A and B we provide further mathematical details and case studies to support our results.

## Model and methods

The Hamiltonian for an anisotropic XY spin-half chain in the presence of the Dzyaloshinskii–Moriya (DM) interaction can be expressed as:

1 $$\begin{aligned} \mathscr {H}&= \sum _{l=1}^{N} \bigg \{J\big [(1+\gamma )\,\sigma _{l}^{x}\sigma _{l+1}^{x}+(1-\gamma )\,\sigma _{l}^{y}\sigma _{l+1}^{y} \nonumber \\&\quad +D\,(\sigma _{l}^{x}\sigma _{l+1}^{y}-\sigma _{l}^{y}\sigma _{l+1}^{x})\big ]-\sigma _{l}^{z}\bigg \}. \end{aligned}$$

where *N* is the total number of spins and $\sigma _{l}^{x}$, $\sigma _{l}^{y}$, $\sigma _{l}^{z}$ are the Pauli matrices for the *l*-th spin. *J* is the coupling constant, and is assumed to be in units of the external magnetic field *B*, with $J=J_{s}/B$, where $J_{s}$ is the actual value of the coupling. The choice to normalize *J* by *B* is made because interesting phenomena in the system occur when it is immersed in an external magnetic field. The anisotropy term is represented by $\gamma$ (with $-1 \le \gamma \le 1$), and *D* is the Dzyaloshinskii–Moriya (DM) interaction factor. This sets the stage for the rest of the paper, particularly for local and quasi-local approaches to metrology. In both cases, we need the reduced density matrices, which are essential for understanding the system’s behavior when only partial accessibility to the system is available. In particular, we are going to consider the thermodynamic limit $N\rightarrow \infty$ and at zero temperature. For a local measurement, the single-spin reduced density matrix $\rho _1$ is obtained by tracing out all spins except one from the total density matrix. The resulting density operator is actually independent of the specific spin and reads

2 $$\begin{aligned} \rho _1 = \frac{1}{2} \left( {\mathbb I}+ \langle \sigma ^{z}\rangle \, \sigma ^z\right) \end{aligned}$$

where $\langle \sigma ^{z}\rangle =\hbox {Tr}[\rho _1\, \sigma ^z]$ denotes the mean magnetization per spin of the system, i.e.

3 $$\begin{aligned} \langle \sigma ^z \rangle = - \frac{1}{\pi } \int _0^{\pi } d\phi \ \frac{\left[ J(\cos \phi - 2D \sin \phi )-1\right] }{\Delta }, \end{aligned}$$

where

4 $$\begin{aligned} \Delta = \sqrt{\left[ J(\cos \phi - 2D \sin \phi )-1 \right] ^2 + J^2 \gamma ^2 \sin ^2 \phi }. \end{aligned}$$

Indeed, the reduced density matrix of a single spin only depends on the local properties of the system: the diagonal elements depend only on the local magnetization, and the reduced density is the same independently on the exact location of the spin, as expected from the translational invariance of the Hamiltonian in Eq. (1).

If we move to two spins, the reduced density matrix includes not only local properties but also the correlations between them. Upon tracing over all the spins except those corresponding to the *j* and *k* sites we have

5 $$\begin{aligned} \rho _2=\hbox {Tr}_{\overline{jk}}(\rho ). \end{aligned}$$

Due to the symmetries inherent in the physical model under analysis, the two-site reduced density matrix has the following *X*-structure^49^

6 $$\begin{aligned} \rho _2 \equiv \rho _2 (r) = \begin{pmatrix} a_+ & 0 & 0 & b_- \\ 0 & c & b_+ & 0 \\ 0 & b_+ & c & 0 \\ b_- & 0 & 0 & a_- \end{pmatrix}, \end{aligned}$$

where the matrix elements $a_{\pm }$, $b_{\pm }$ and *c* are given by

7 $$\begin{aligned} a_{\pm }&=\frac{1}{4}\big [ 1 \pm 2 \langle \sigma ^z \rangle +\langle \sigma ^z_j \sigma _{j+r}^z \rangle \big ], \nonumber\\ b_{\pm }&=\frac{1}{4}\big [ \langle \sigma _j^x \sigma _{j+r}^x \rangle \pm \langle \sigma _j^y \sigma _{j+r}^y \rangle \big ], \nonumber \\ c&=\frac{1}{4} \big [ 1 - \langle \sigma _j^z \sigma _{j+r}^z \rangle \big ]. \end{aligned}$$

The quantity $\langle \sigma ^{z}\rangle$ is given in Eq. (3) whereas

$$\begin{aligned} \langle \sigma _j^\alpha \sigma _{j+r}^\alpha \rangle \equiv S^\alpha _r\, \quad \alpha =x,y,x \end{aligned}$$

denote the correlations between the components of the two spins. Due to the translational invariance of the Hamiltonian in Eq. (1), these correlation functions do depend only on the distance *r* between the two spins. The spin-spin correlation functions $S^x_r$ and $S^y_r$ can be computed from the determinant of Toeplitz matrices^50,51^ as

8 $$\begin{aligned} S^x_r = \begin{vmatrix} G_{-1}&G_{-2}&\dots&G_{-r} \\ G_{0}&G_{-1}&\dots&G_{-r+1} \\ \vdots&\vdots&\ddots&\vdots \\ G_{r-2}&G_{r-3}&\dots&G_{-1} \end{vmatrix} \, \end{aligned}$$

9 $$\begin{aligned} S^y_r = \begin{vmatrix} G_{1}&G_{0}&\dots&G_{-r+2} \\ G_{2}&G_{1}&\dots&G_{-r+3} \\ \vdots&\vdots&\ddots&\vdots \\ G_{r}&G_{r-1}&\dots&G_{1} \end{vmatrix} \, \end{aligned}$$

in which *r* is the distance between the two spins. For the z-direction we have

10 $$\begin{aligned} S^z_r = {\langle {\sigma }_{i}^z \rangle }^2 - G_r G_{-r} \, \end{aligned}$$

where

11 $$\begin{aligned} G_k&= - \frac{1}{\pi } \int _0^{\pi } d\phi \ \frac{2 \cos (\phi k)}{\Delta } \left[ J(\cos \phi - 2D \sin \phi )-1\right] \nonumber \\&\quad + \frac{\gamma }{\pi } \int _0^{\pi } d\phi \ \frac{2 J \sin (\phi k)}{\Delta } \sin {\phi }. \end{aligned}$$

The integrals in Eqs. (3) and (11) cannot be evaluated analytically and therefore we calculate them numerically. Moreover, given the structure of the Hamiltonian in Eq. (1), we study the phenomenology of the system for $J \ne 0$.

## Quantum and classical Fisher information theory

As the aim of this work is to focus on metrology under the assumption of partial system accessibility, it is crucial to introduce key quantities adopted to characterize the system. The study is based on the analysis of the quantum and classical Fisher information, which quantify the ultimate bounds to precision in the estimation of system parameters^52–55^. The QFI is intrinsically related to the geometry of the manifold of quantum states, i.e. the Bures distance^56,57^ as

12 $$\begin{aligned} d^{2}_{B}(\rho _{\lambda },\rho _{\lambda +d\lambda }) = \frac{1}{4} H(\lambda ) d\lambda ^{2}, \end{aligned}$$

where $H(\lambda )$ is referred to as the quantum Fisher information (QFI)^58–60^ associated to a parameter $\lambda$ of the Hamiltonian. Since quantum phase transitions are described as an abrupt change in the ground state of a many-body system due to the variation of a physical parameter, there is a deep relation among phase transitions and the divergences in the QFI^2,61–65^. In particular, we expect critical spin chains to provide enhanced precision for those values of the parameters corresponding to quantum phase transitions, where $H(\lambda )$ diverges. The QFI is itself also related to the fidelity^58–60^ and may be evaluated as

13 $$\begin{aligned} H(\lambda ) = \hbox {Tr}[ \rho _{\lambda }\mathscr {L}_{\lambda }^{2} ], \end{aligned}$$

where $\mathscr {L}$, the symmetric logarithmic derivative, is related to the variation with respect to a parameter $\lambda$ of the density matrix $\rho$ as

14 $$\begin{aligned} \partial _{\lambda } \rho _{\lambda } = \frac{1}{2} \{ \mathscr {L}_{\lambda }, \rho _{\lambda } \}, \end{aligned}$$

where $\{ \, , \ \}$ indicates the anti-commutator. The QFI sets a bound on the variance of any (unbiased) estimator used to infer the value of the parameter of interest from data, as

15 $$\begin{aligned} V(\lambda ) \ge \frac{1}{MH(\lambda )}, \end{aligned}$$

where *M* is the number of measurements.

According to the partial accessibility hypothesis we focus on the one and two spin reduced density matrix presented in “Model and methods”. The single-spin density matrix in Eq. (2) is diagonal in the basis of $\sigma ^z$, i.e. $\rho _1= p |0\rangle \langle 0|+ (1-p) |1\rangle \langle 1|$ with $p = \frac{1}{2} (1+ \langle \sigma ^z \rangle )$ and thus the QFI may be easily evaluated as

16 $$\begin{aligned} H(\lambda ) = \frac{(\partial _\lambda p)^2}{(1-p) p} = \frac{(\partial _\lambda \langle \sigma ^z \rangle )^2}{1-\langle \sigma ^z \rangle ^2}. \end{aligned}$$

For the two-spin reduced density matrix of Eq. (6) the QFI reads as follows^66^

17 $$\begin{aligned} H_r(\lambda )&= \frac{1}{a_0} \left[ \frac{(\sum _{jk}\eta _{jk} a_j \partial _\lambda a_k)^2}{\sum _{jk}\eta _{jk} a_j a_k} - \sum _{jk} \eta _{jk} \partial _\lambda a_j\, \partial _\lambda a_k \right] \nonumber \\&\quad + \frac{1}{b_0} \left[ \frac{(\sum _{jk}\eta _{jk} b_j \partial _\lambda b_k)^2}{\sum _{jk}\eta _{jk} b_j b_k} - \sum _{jk} \eta _{jk} \partial _\lambda b_j\, \partial _\lambda b_k \right] \nonumber \\&\quad + \frac{(\partial _\lambda a_0)^2}{a_0} + \frac{(\partial _\lambda b_0)^2}{b_0}\ . \end{aligned}$$

where

18 $$\begin{aligned} a_0&= \frac{1}{2}\left( 1 + S_r^z\right) ,\, a_1 =\frac{1}{2}\left( S_r^x -S_r^y\right) ,\, a_2 = 0,\, a_3 = \langle \sigma ^z \rangle \,,\nonumber \\ b_0&= \frac{1}{2}\left( 1 - S_r^z\right) ,\, b_1 =\frac{1}{2}\left( S_r^x + S_r^y\right) ,\, b_2 = 0,\, b_3 = 0\,. \end{aligned}$$

and $$\eta = \hbox {Diag}\{1,-1,-1,-1\}$$.

The QFI provides an upper bound to the Fisher information (FI) of any possible measurement that may be performed on the system in order to infer the value of the parameter. The FI itself is defined as

19 $$\begin{aligned} F(\lambda ) = \sum _{x} p(x \vert \lambda ) \big [ \partial _{\lambda } \log p(x \vert \lambda ) \big ]^{2}. \end{aligned}$$

and since the density matrix $\rho _1$ is diagonal in the *z*-basis, we have that for local magnetization measurement $F(\lambda )=H(\lambda )$. For a two spin magnetization measurement the FI is given by

20 $$\begin{aligned} F(\lambda )= \frac{1}{a_+} \left( \frac{\partial a_+}{\partial \lambda } \right) ^2 + \frac{2}{c} \left( \frac{\partial c}{\partial \lambda } \right) ^2 + \frac{1}{a_-} \left( \frac{\partial a_-}{\partial \lambda } \right) ^2, \end{aligned}$$

and since $\rho _2$ is non-diagonal in the *z*-based, the Fisher information is lower than its quantum counterpart, i.e.,

21 $$\begin{aligned} F(\lambda )\le H(\lambda ). \end{aligned}$$

When the system is defined by more than one parameter $\varvec{\lambda }=\{{\lambda }_{1},{\lambda }_{2},\ldots ,{\lambda }_{n}\}$, as in Eq. (1), the Bures distance generalizes to a metric as

22 $$\begin{aligned} d^{2}_{B}(\rho _{\varvec{\lambda }},\rho _{\varvec{\lambda }+d\varvec{\lambda }}) = \frac{1}{4} H_{\mu \nu }(\varvec{\lambda }) d\mu d\nu , \end{aligned}$$

where $H_{\mu \nu }(\varvec{\lambda })$ are the elements of the so-called quantum Fisher information matrix (QFIM), defined as

23 $$\begin{aligned} H_{\mu \nu }(\varvec{\lambda }) = \frac{1}{2}\,\hbox {Tr}[\rho _{\varvec{\lambda }} \left( \mathscr {L}_{\mu } \mathscr {L}_{\nu }+ \mathscr {L}_{\nu } \mathscr {L}_{\mu } \right) ], \end{aligned}$$

where $\mathscr {L}_{\mu }$ and $\mathscr {L}_{\nu }$ are the symmetric logarithmic derivatives associated to the components $\lambda _{\mu }$ and $\lambda _{\nu }$ of the vector $\varvec{\lambda }$ respectively. For the spin chain Hamiltonian (Eq. (1)) the parameters are the coupling constant *J*, the anisotropy parameter $\gamma$ and the DM interaction factor *D*, so in this case $\varvec{\lambda }=\{J,\gamma ,D\}$.

The inverse of the QFI matrix provides a lower bound on the covariance matrix $\varvec{V}$ of the set of estimators, i.e., ${\varvec{V}}_{\mu \nu }=\langle \lambda _{\mu } \lambda _{\nu }\rangle -\langle \lambda _{\mu } \rangle \langle \lambda _{\nu }\rangle$, which reads

24 $$\begin{aligned} \varvec{V} \varvec{(\lambda )} \ge \frac{1}{M} {\varvec{H}}^{-1}\varvec{(\lambda )}. \end{aligned}$$

By introducing a positive, real matrix of dimension $n \times n$, i.e. the weight matrix *W*, a scalar bound may be obtained. In the following we consider simple choice $W=\mathbb {1}$, obtaining a relation in the form

25 $$\begin{aligned} \hbox {Tr} \left[ {\varvec{WV}} \right] = \sum _{\mu } \hbox {Var}(\lambda _{\mu }) \ge \frac{1}{M} \hbox {Tr} \left[ {\varvec{H}}^{-1}\varvec{(\lambda )} \right] \, \end{aligned}$$

i.e. a lower bound on the sum of the variances associated to the parameters contained in the vector $\varvec{\lambda }$.

A fundamental tool in multi-parameter estimation is the so called Uhlmann matrix, whose elements are defined as

26 $$\begin{aligned} {\varvec{U}}_{\mu \nu }\varvec{(\lambda )} = \hbox {Tr}\left[ \rho _{\varvec{\lambda }}\frac{\mathscr {L}_{\mu }\mathscr {L}_{\nu }-\mathscr {L}_{\nu }\mathscr {L}_{\mu }}{2}\right] . \end{aligned}$$

A vanishing Uhlmann matrix means that the parameters may be jointly estimated with the same precision achievable from their separate estimations. On the other hand, if ${\varvec{U}}_{\mu \nu }\varvec{(\lambda )}\ne 0$ there is an intrinsic additional noise of quantum origin in the joint estimation of $\lambda _{\mu }$ and $\lambda _{\nu }$, due to the non commutativity of the corresponding SLDs.

Finally, another relevant feature associated to the Fisher matrix is its determinant. It is a measure of the *degree of sloppiness of the system* that quantify how strong is the dependence of the system on a combination of the components of $\varvec{\lambda }$ rather then on its components separately. When the Fisher matrix is singular (i.e. $det[\varvec{H(\lambda )}]$=0), the statistical model is referred to as *sloppy*, and this means that the true parameters that describe it are combinations of the original parameters $\{{\lambda }_{1},{\lambda }_{2},\ldots ,{\lambda }_{n}\}$. For this reason the closer is $det[\varvec{H(\lambda )}]$ to zero the higher is the degree of sloppiness of the system.

## Local measurements

**Figure 1:**
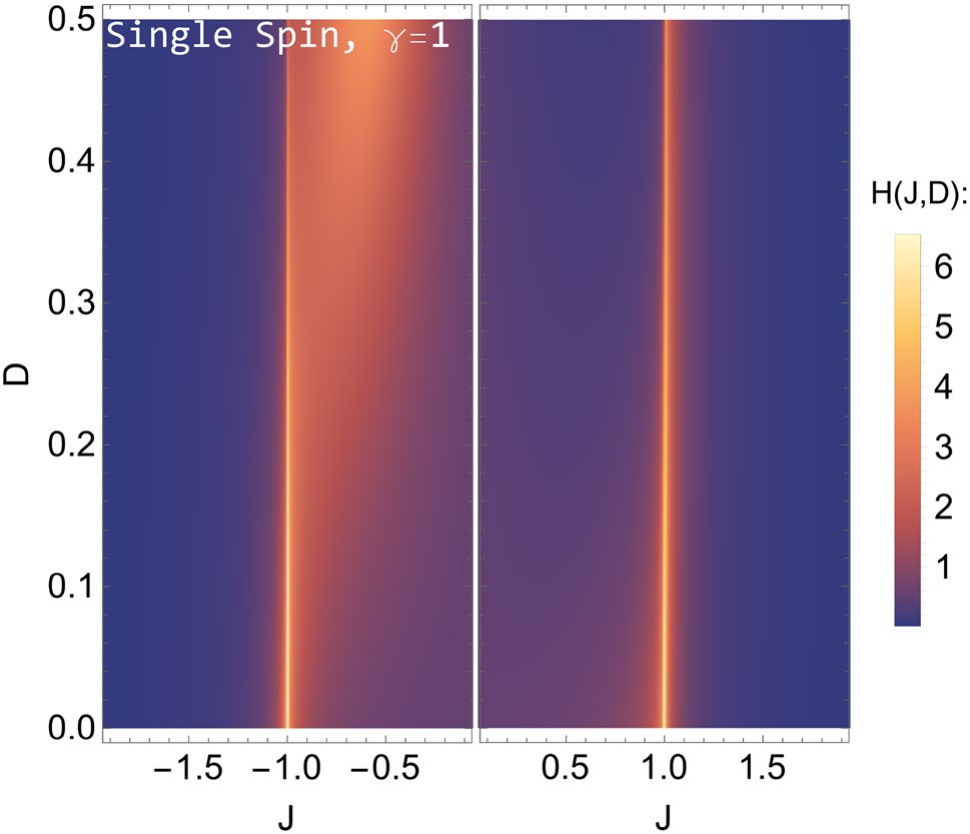
(Quantum) Fisher information of *J* for a single spin reduced density matrix of an anisotropic Heisenberg XY spin chain, with Dzyaloshinskii–Moriya interaction. The anisotropy parameter value is fixed to $\gamma =1$ (Ising model). Here we can see the dependence on the Hamiltonian parameters *J* and *D*, for $J\ne 0$. For $J=\pm 1$, *H*(*J*) diverges $\forall D$. This allows to distinguish the two phases of the system from a local measurement.

We start our analysis from the information that can be extracted by accessing a single spin. In this case, the best measurement is the magnetization along the direction of the external field since, as mentioned above, its FI equals the QFI. In Fig. 1 we show the QFIM element $H_{JJ}(J,D)$ associated to the coupling constant of the spin chain as a heat-map, as a function of *J* and *D* for a fixed value of $\gamma =1$ (Ising model). We notice a divergence for $J=\pm 1$, as it happens for the QFI of a collective measurement^61^. The parameter *D* affects the behavior of $H_{JJ}$ mainly for $J<0$. As *D* increases, the range of *J* in which $H_{JJ}$ is large increases too, whereas the value at the peak in $J=-1$ decreases. Results for $\gamma \ne 1$ are similar. These results show how that due to the Hamiltonian properties, even a local measurement detects the phase transitions of the spin chain and therefore may be exploited in a metrological protocol. This can be intuitively understood by looking at the structure of the single spin reduced density matrix. Since it depends only on the mean magnetization of a spin, the metrological results should reflect this characteristic, and bring the same properties as the ones related to a measurement that depends on the global magnetization of the whole system. The precision should be however compared to that achievable by quasi local measurements, where correlations usually enhance the information that may be extracted. This will be the subject of the next section.

## Quasi local measurements and the role of correlations

Let us now consider estimation protocols based on measuring two spins of the chain. In this case, besides local magnetization, the results of the measurement are also influenced by the correlations between the spins. To begin, it is useful to start from the limit of infinitely distant spins. This because for non-interacting spins the (Q)FI is just twice the single-spin (Q)FI, and this case may be used as a reference to understand whether correlations are beneficial or detrimental for quantum metrology. Since the Hamiltonian involves interactions between nearest neighbors, we may expect the correlation to vanish by increasing the distance between the two measured spins. Indeed, we found numerically that $|G_{r}|<1$ $\forall r$ for the entire range values of *J*, $\gamma$ and *D* used in this work. Furthermore, we found that the functions $G_{r}$ goes to zero as 1/*r*.

27 $$\begin{aligned} G_{r} \simeq -G_{-r} \propto \frac{1}{r} \ \ \hbox {for} \ \ r \rightarrow \infty , \end{aligned}$$

and, in turn,

28 $$\begin{aligned} \langle \sigma _i^x \sigma _{i+r}^x \rangle&\propto \frac{1}{r} \qquad \langle \sigma _i^y \sigma _{i+r}^y \rangle \propto \frac{1}{r} \end{aligned}$$

29 $$\begin{aligned} \langle \sigma _i^z \sigma _{i+r}^z \rangle&\simeq {\langle {\sigma }_{i}^z \rangle }^2 \,. \end{aligned}$$

Correspondingly, the two-spin reduced density matrix (6) assumes the diagonal form

30 $$\begin{aligned} \rho _2(\infty ) = \hbox {Diag}[a_+,c,c,a_-]. \end{aligned}$$

This structure implies that the classical FI of magnetization equals the QFI and is given by

31 $$\begin{aligned} H(\lambda ) = F(\lambda ) = 2\, \frac{{\partial _\lambda \langle \sigma ^z \rangle }^2}{{1-\langle \sigma ^z \rangle }^2}, \end{aligned}$$

i.e., in this case, measuring the magnetization allows us to extract the entire information present in the system, which is twice the value of the single spin case. This is true $\forall \lambda \in \{J,\gamma , D\}$. In turn, this results follows directly from the symmetry of the model since, due to the translation invariance of the Hamiltonian in Eq. (1) every spin of the model contributes equally to the (Q)FI in the absence of correlations.

**Figure 2:**
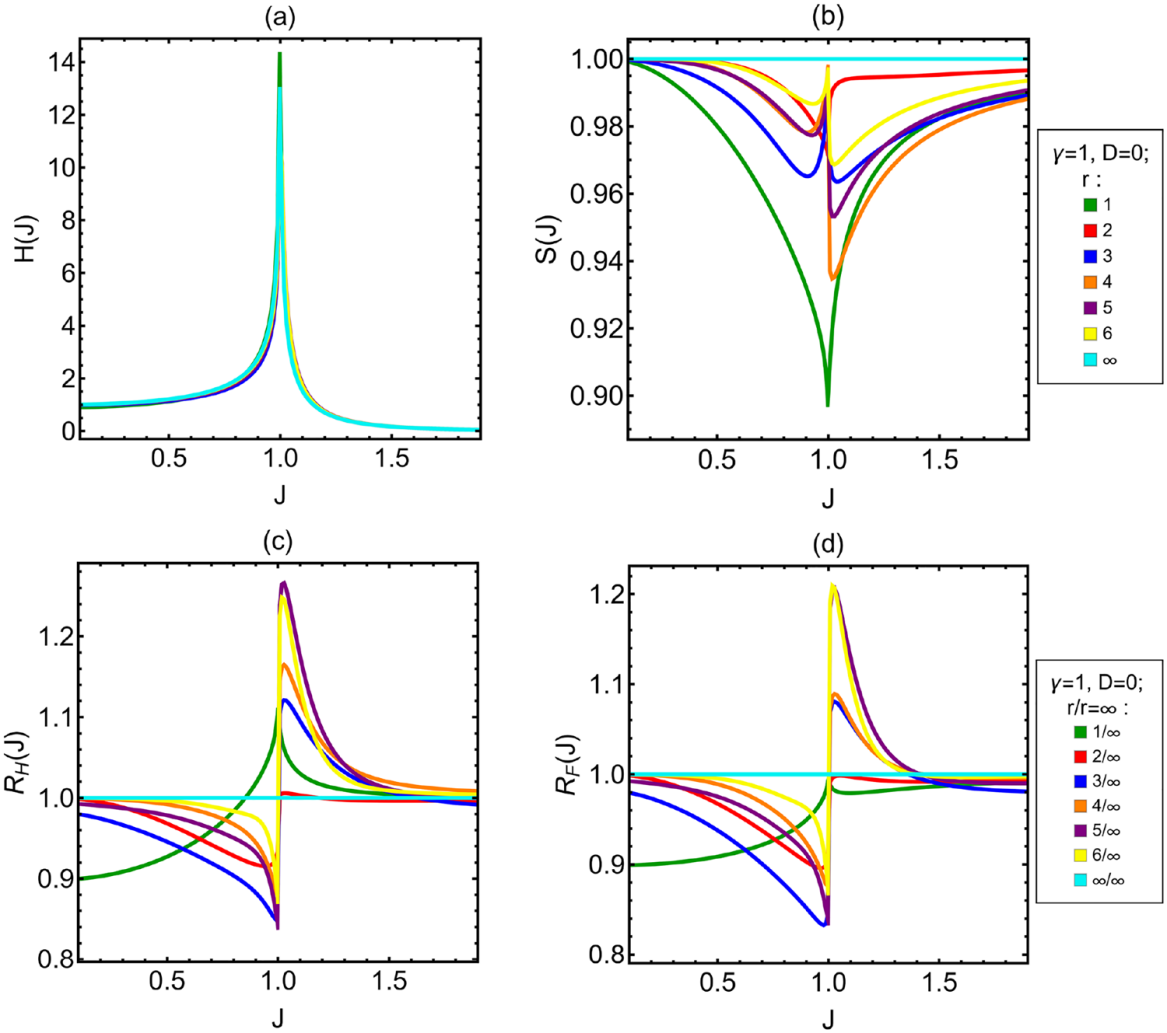
(**a**) QFI of coupling constant *J* for different values of *r*. (**b**) Saturation associated to *J*. *S*(*J*) is always above 0.9. In the limit of infinitely distant spins $H(J)=F(J)$. (**c**,**d**) Ratios between the (Q)FI for different distances and the limiting QFI of infinitely distant neighbors $R_{(H)F}(J)$. For *H*(*J*) and *F*(*J*) the optimal distance depends differently on *J*. The anisotropy parameter is $\gamma =1$ and the DM factor is $D=0$. Different curves and colors are associated to different values of the distance between the two spins measured *r*.

We now move to the study of the FI and QFI as a function on the distance between the measured spins, going beyond the first nearest neighbors metrology, looking for optimal configurations in the different realizations of the model. Let us start from the Ising model without DM interaction (i.e. the anisotropy parameter is set to $\gamma =1$ and the DM interaction factor to $D=0$). In Fig. 2, we show the QFI *H*(*J*) as a function of *J* for a distance between the spins measured $r\in \{1,2,3,4,5,6,\infty \}$. We show *H*(*J*) only for positive *J*, because when $D=0$ the QFI is even in *J*, $H(J)=H(-J)$. This directly arise from the structure of the Hamiltonian in Eq. (1), because when $D=0$ the difference between $\mathscr {H}(J)$ and $\mathscr {H}(-J)$ is just a global phase. The behavior of *H*(*J*) is qualitatively the same regardless the distance. In particular all curves show a divergence for $J=\pm 1$. This implies that *H*(*J*) is strongly affected by the phase transition from the ferromagnetic to the paramagnetic phase of the spin chain and the system is an excellent sensor in this region. To understand if the phase transition is detectable in practice, we also studied the FI *F*(*J*) of magnetization measurement. The general behavior of *F*(*J*) and *H*(*J*) is very close, with the same symmetry, $F(J)=F(-J)$ and the same divergences at $J=\pm 1$, though they differ quantitatively. For this reason, we show the ratio between these two quantities, termed saturation and defined as

32 $$\begin{aligned} S(J)=\frac{F(J)}{H(J)} \end{aligned}$$

In the top right panel of Fig. 2, we show *S*(*J*) for different *r*. As mentioned above, $S(J)=1$ for $r\rightarrow \infty$, and it remains very high ($S(J) \gtrsim 0.9$) for the entire range of distances we explored ($1\le r \le 6$). We conclude that is feasible to extract a large part of information from the Ising spin chain through a magnetization measurement. To understand if there is an optimal distance that maximizes the QFI and the FI we look at the ratios between the (Q)FI of the different neighbors and (Q)FI in the limit of infinitely distant spins, i.e. the quantities

33 $$\begin{aligned} R_{H}(J)=\frac{H(J;r)}{H(J;\infty )} ; \; R_{F}(J)=\frac{F(J;r)}{F(J;\infty )}, \end{aligned}$$

which is shown in the lower panels of Fig. 2. From the lower left panel (i.e. QFI ratios), we see that the optimal distance between the measured spins depends on the value of *J* itself, and in turn on the value of the external magnetic field. In the ferromagnetic phase (i.e. $J<1$) and for $0<J \lesssim 0.9$, the optimal choice is to measure very distant spins ($r \rightarrow \infty$), whereas in the region from $J\approx 0.9$ to $J=1$ we have the opposite, i.e. the optimal choice is to measure two nearest neighboring spins ($r=1$). In the paramagnetic phase, from $J=1$ to a value close to $J\approx 1.25$ the optimal distance is $r=5$, while for higher values of *J* the optimal distance is $r=4$. Concerning the FI, we see from the lower right panel of Fig. 2 that also in this case the optimal distance depends on *J*. In the paramagnetic phase, in the region close to $J=1$, upon the distances analyzed, the optimal distance is $r=6$, then up to a value close to $J=1.4$ it becomes $r=5$ and after this value it goes again to $r\rightarrow \infty$. In this phase, close to $J=1$, we can notice an even/odd effect such that the curves for $r=3$ and $r=4$ and the curves for $r=5$ and $r=6$ are almost paired. A similar effect is present also in the ratios for the QFI, but less marked. Comparing the lower panels of Fig. 2, another emerging feature is that the optimal distances to optimize FI and QFI are in general different for a given value of *J*.

**Figure 3:**
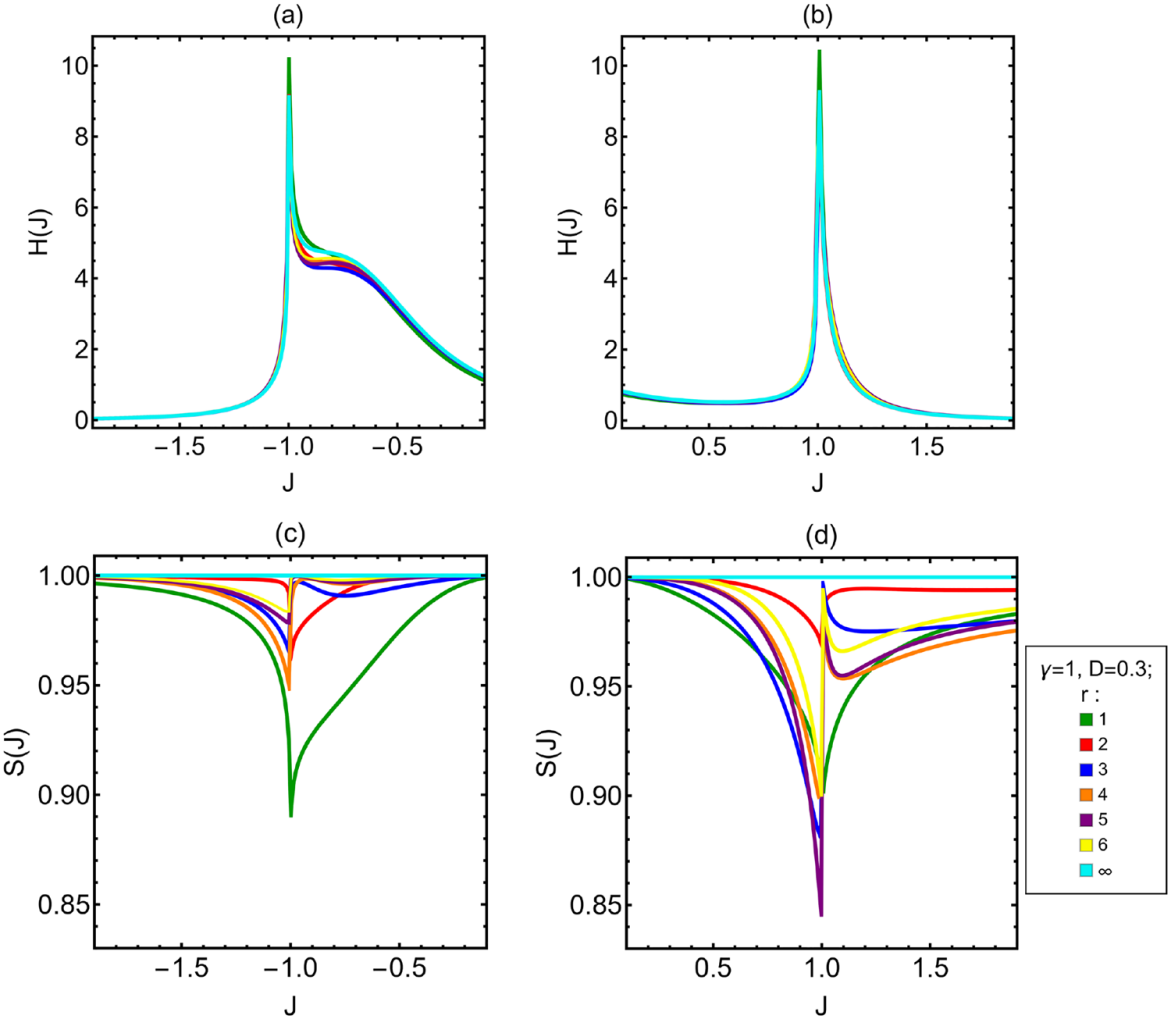
(**a**,**b**) QFI of the coupling constant *J*. (**c**,**d**) Saturation *S*(*J*) associated to the coupling constant *J*. In the limit of infinitely distant spins $H(J)=F(J)$. All the plots in figure are as *J* varies. The anisotropy parameter is $\gamma =1$ and the DM factor is $D=0.3$. The different curves are associated to the different values of the distance between the two spins measured *r*.

**Figure 4:**
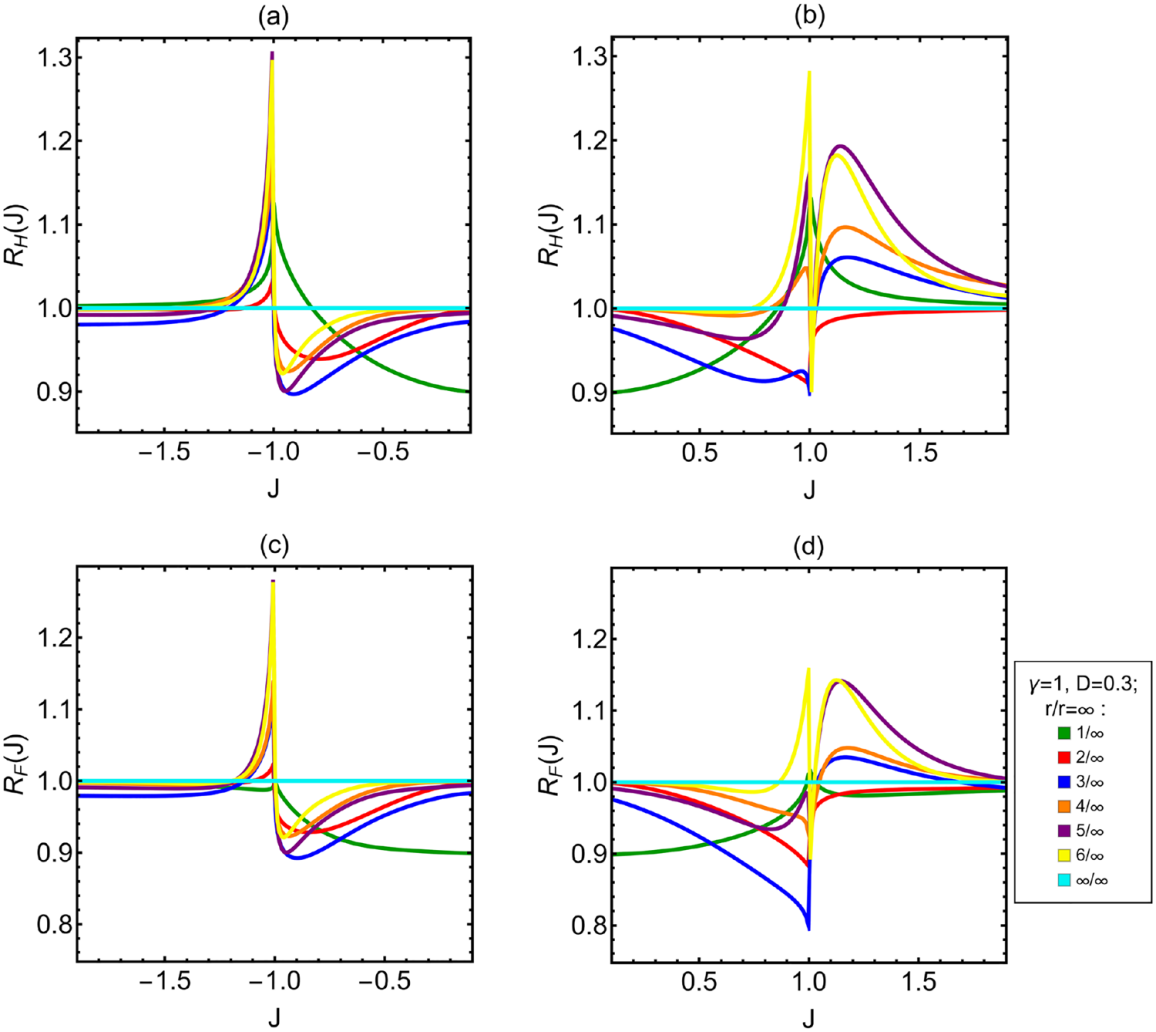
(**a**,**b**) Ratios between the QFI for two spins at distance *r* and QFI in the limit of infinitely distant neighbors, $R_{H}(J)$ as a function of *J* and for different *r*. As it was without DM interaction, also in this case the optimal distance depends on *J*. (**c**,**d**) Ratios between the FI of the different neighbors and FI in the limit of infinitely distant neighbors, $R_{F}(J)$ as a function of *J* and for different *r*. The anisotropy parameter is $\gamma =1$ and the DM factor is $D=0.3$.

After the analysis of the Ising model, we now study the impact of the DM interaction. For the sake of concreteness, we focus on the specific case of $\gamma =1$ and $D=0.3$. Notice also that due to the structure of the Hamiltonian, what matters is the relative sign between *J* and *D* and the phenomenology observed for positive (negative) *J* and $D>0$ is the same observed for negative (positive) *J* and $D<0$. Hence, for $D\ne 0$ we have to analyze the behavior of the QFI for positive and negative *J*, because we could expect it to be different. In the top panels of Fig. 3, we show the *H*(*J*) as a function of *J* for different spin distances $r\in \{1,2,3,4,5,6,\infty \}$. The first thing to notice is that the QFI is no longer even in *J*, whereas the general behavior of the curves is nearly independent on *r*, as it happens for $D=0$. The two divergences at $J= \pm 1$ are still present, but in this case a bump appears on the right of the divergence at $J=-1$. These features appear also for the FI and in turn, independently on *D*, the FI and the QFI are very close. These properties are reflected in the plot of the saturation *S*(*J*), in the lower panels of Fig. 3, which is always above $S(J)\approx 0.84$. In order to find for the optimal spin distance in the presence of DM interaction, we look at the ratios between the QFI (FI) for different spin distances and the that associated to infinite distance (i.e. $R_{H(F)}(J)$), see the top panels of Fig. 4. Also in presence of DM interaction the optimal distance depends on *J*. In the region around the value $J\approx -2$, the optimal distance is $r=1$, then for a very short interval $r=4$ and after up to $J=-1$ it is $r=5$. In the ferromagnetic phase, from $J=-1$ to a value close to $J\approx -0.85$ the optimal distance is $r=1$ and then it is $r=\infty$ up to another value of *J* close to $J=0.75$. After that the optimal distance is $r=6$ until $J=1$. For $J>1$ the optimal distance is $r=1$ up to a value of *J* close to $J=1.05$ then it is $r=5$. Comparing these ratios to those for Ising model (Fig. 2), we can see two interesting features arising from the presence of the DM interaction. The first one is that the peaks associated to $r=3$, $r=4$, $r=5$ and $r=6$ in the paramagnetic phase, close to $J=1$, are higher respect to the case without DM interaction for $J<0$ and lower than the case without DM interaction for $J>0$. This is a general trend observed for all the values of $\gamma$ and *D* studied (see also Appendix A). In particular, the higher is the value of *D*, the higher is the increase respect to the case $D=0$. The other interesting feature is that for $J<0$ the curves associated to $r=4$, $r=5$ and $r=6$ are higher than the curve for $r=\infty$. Concerning the ratios of FI, the lower panels of Fig. 4 show that starting from $J\approx -2$ the optimal distance is $r=\infty$ up to a value of *J* close to $J\approx -1.2$ then until $J=-1$ it is $r=5$. In the ferromagnetic phase, the optimal distance is $r=\infty$ up to a value of *J* close to $J=0.85$ after that it is $r=6$ until the phase transition. In the paramagnetic phase, very close to $J=1$ the optimal distance is $r=1$, then it almost immediately changes to $r=6$, whereas approaching $J=1.1$ it becomes $r=5$. Comparing these ratios with those of the the Ising model, we see the same feature for the peaks in the paramagnetic phase that is observed for the QFI. Also for the ratios of the FI with $D=0.3$ in paramagnetic phase there is a loss in the peaks close to $J=1$ for the curves associated to $r=3$, $r=4$, $r=5$ and $r=6$, respect the analogue curves for $D=0$. Instead there is a gain for the peaks close to $J=-1$. Another interesting feature is that, as for the QFI, in the FI associated to the ferromagnetic phase when $J>0$ the optimal distance close to the phase transition is $r=6$, not $r=\infty$ as it is for the Ising model without DM interaction.

From the comparison between the results with and without DM interaction, few general conclusions may be drawn. The first is that the optimal distance for the FI and the QFI are, in general, different. This could be expected from the fact that, except for the limit of infinitely distant spins, the QFI depends on all the correlation functions $\langle \sigma _i^x \sigma _{i+r}^x \rangle$, $\langle \sigma _i^y \sigma _{i+r}^y \rangle$ and $\langle \sigma _i^z \sigma _{i+r}^z \rangle$, whereas the FI depends only on $\langle \sigma _i^z \sigma _{i+r}^z \rangle$. In addition, we have that the optimal distance for both the FI and the QFI depends on all the Hamiltonian parameters, *J*, $\gamma$ and *D*, therefore we can not define a universal optimal distance for metrological applications.

## Multi-parameter estimation for quasi local measurements

After having analyzed the bounds to precision for the coupling constant *J*, we now move to study the impact of the distance *r* between the measured spins on the precision of all Hamiltonian parameters. First of all, we notice that being the density matrix of the system a real X-state the Uhlmann matrix vanishes (see Appendix B). Actually, the symmetric logarithmic derivatives for the different parameters do not commute, but they do *weakly*, i.e. the commutators have vanishing expectation value, such that the system is *asymptotically classical*^67^. As a consequence, it is possible to perform the joint estimation of the Hamiltonian parameters *J*, $\gamma$ and *D* without any additional intrinsic noise of quantum origin.

**Figure 5:**
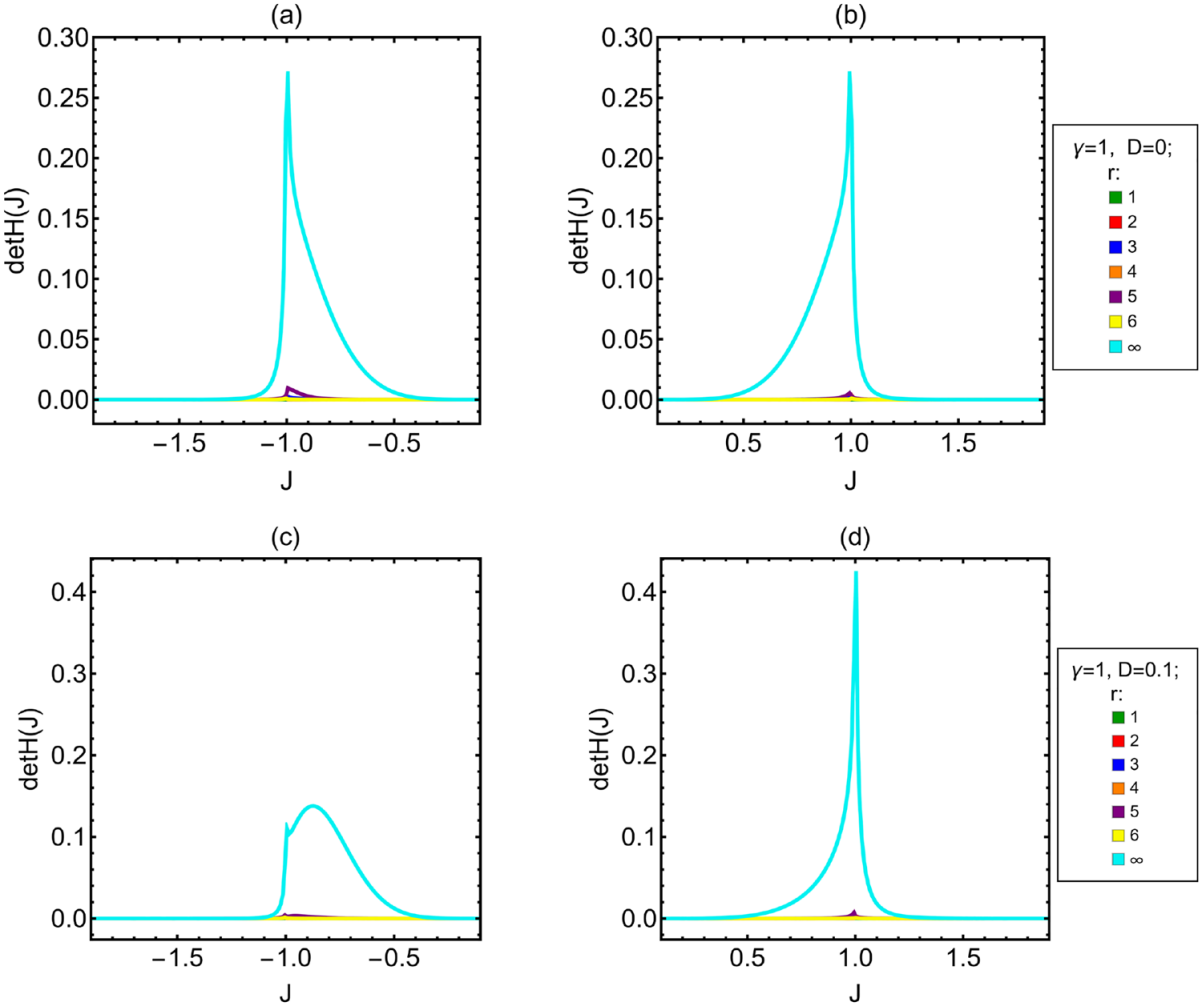
Determinant of the quantum Fisher matrix $\hbox {det}H$ as a function of *J*. (**a**,**b**) Ising model without DM interaction. (**c**,**d**) Ising model with DM interaction and $D=0.1$. Different curves and colors are associated to different values of the distance *r* between the two measured spins; $r=\infty$ correspond to the limit of infinitely distant spins.

Let us now analyze the degree of sloppiness of the model, i.e. whether the state of the system depends on *J*,$\gamma$ and *D* separately or only on a combination of them. This may investigated by looking at the determinant of the QFI matrix, see Fig. 5.

It is immediately clear that, except close to the phase transitions $(J=\pm 1)$, for both $D=0$ and $D=0.1$ the determinant is negligible when $1\le r \le 6$. This means that, away from the phase transitions, the system exhibits a high degree of sloppiness, so that we can consider it sloppy for any practical purposes. Even close to $J=\pm 1$ such degree is still very high for finite values of *r*, while for $r=\infty$, there are wide intervals of *J* for which the determinant is significantly different from zero. In these regions the degree of sloppiness is much lower than for the other neighbors, therefore $r=\infty$ is the most suitable choice for the practical joint estimation of the Hamiltonian parameters. This behavior holds regardless of the value of *D*, which has a weak effect on the specific position and width of the intervals but not on their presence. This suggests that the correlations among the measured spins influence the degree of sloppiness of the system, and this influence can be so strong to decrease the number of effective parameters of the system. For infinitely distant spins the reduced density matrix is diagonal and the QFIM is not affected by correlations (see Eq. (2)), and the intervals of *J* for which the system is not sloppy are significantly wider. Nevertheless there are still regions in which the determinant is nearly zero also for $r=\infty$, and we conclude that the degree of sloppiness does not depend only on the correlations among the spins, but it is a characteristic of the model itself.

**Figure 6:**
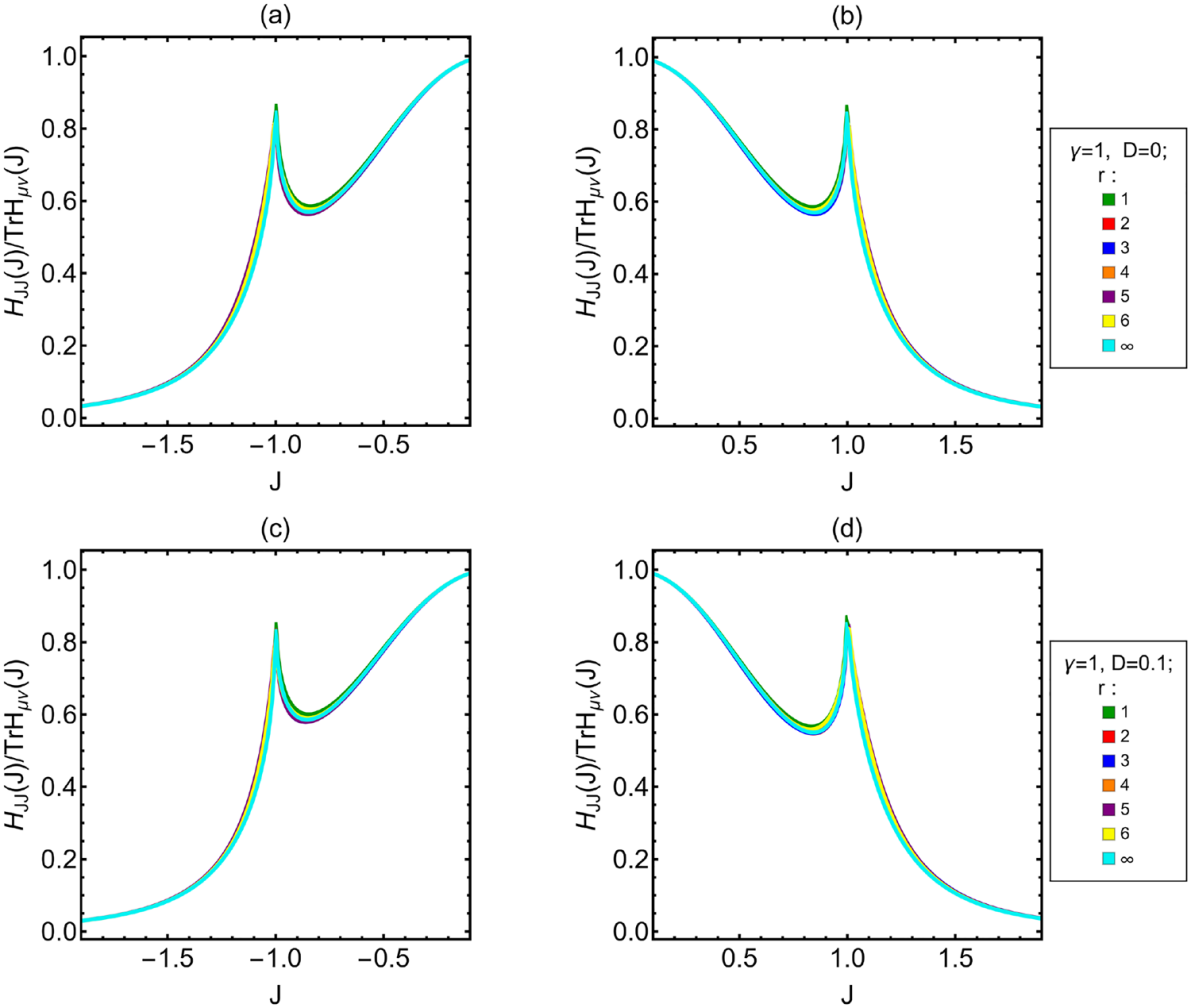
$H_{JJ}/$Tr[*H*] as *J* varies. (**a**,**b**) Ising model without DM interaction. (**c**,**d**) Ising model with $D=0.1$.– Different curves are associated to different distances between the two spins measured *r*. $r=\infty$ correspond to the limit of infinitely distant spins.

Since the sloppiness of the system is large, one may wonder which is the relevant parameter governing the behavior of the system. To this aim it is helpful to analyze the ratio between the QFI of, say, the coupling constant *J* and the trace of the entire QFI. In Fig. 6, we show the ratio $H_{JJ}/$Tr[*H*] as a function of *J* for the different values of *r*, either with or without DM interaction. These plots show that $H_{JJ}$ represents the main contribution to the trace in the most part of the *J* domain. Where it is not, the trace value is very low. This implies that *J* carries the most relevant part of the information about the system behavior, and justify our choice to focus our single parameter study on the QFI and the FI associated to *J*.

The last step of our work is to study the lower bound to precision for the joint estimation of the Hamiltonian parameters. As explained in “Quantum and classical Fisher information theory”, this bound is provided by the trace of the inverse of the of Tr$[H^{-1}]$ as a function of *J* and different *r*, either with and without DM interaction.

**Figure 7:**
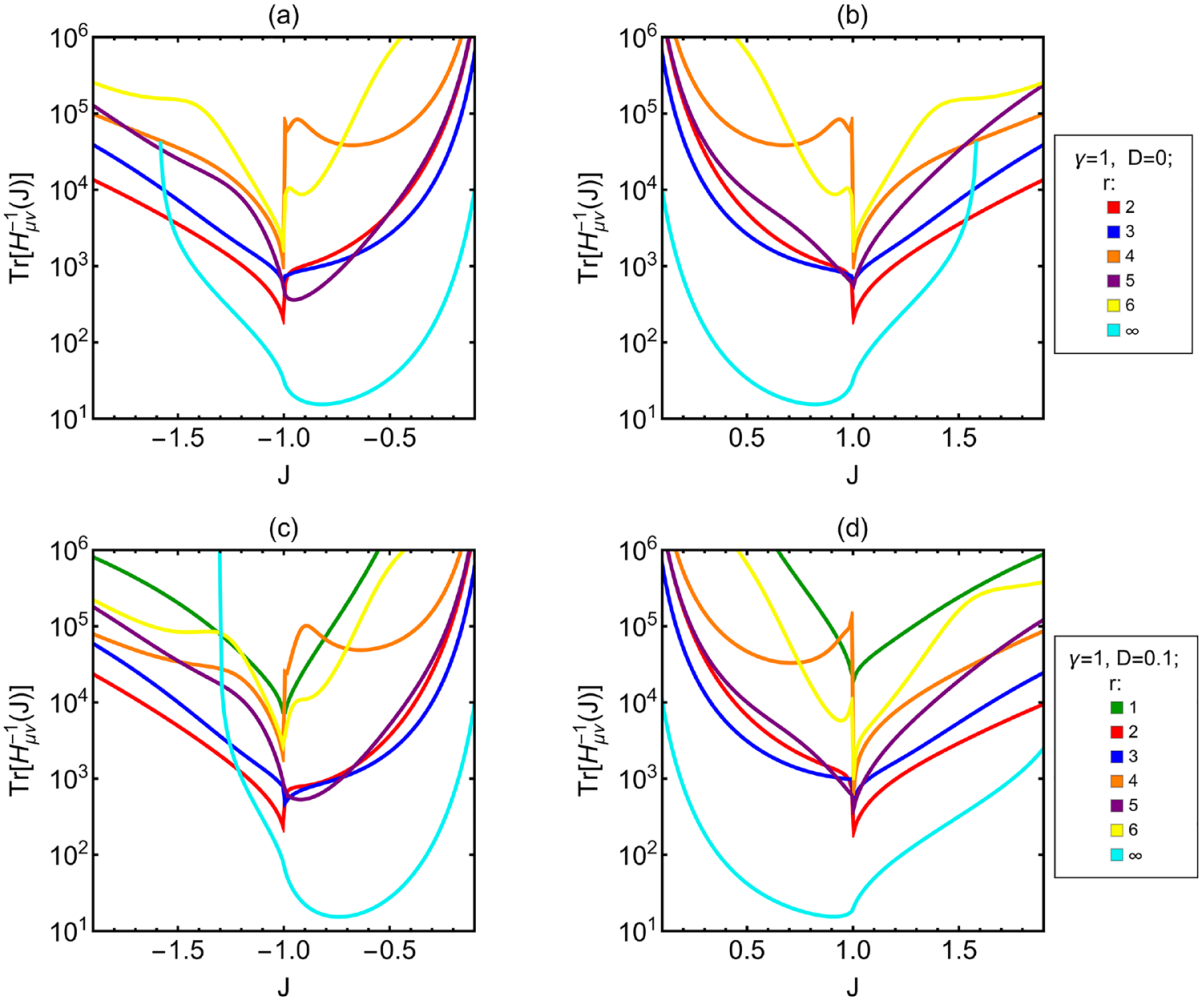
Logplot of Tr$[H^{-1}]$ as a function of *J*. (**a**,**b**) Ising model without DM interaction. (**c**,**d**) Ising model with D = 0.1. Different curves are associated to different distances between the two spins measured *r*. $r=\infty$ correspond to the limit of infinitely distant spins.

For the Ising model without DM interaction the bound associated to $r=1$ is not present and the bound associated to $r=\infty$ disappear for $|J| \gtrsim 1.65$. These facts depends on the behavior of the determinant, which is too low in these cases to invert numerically the Fisher matrix. From Fig. 7 we can see how there is no monotonicity respect *r* either for $D=0$ or $D=0.1$. The most relevant feature in these plots is the order of magnitude for the different neighbors. In the ferromagnetic phase ($|J|<1$) of the Ising model without DM interaction, the difference between the bound for $r=\infty$ and the bounds associated to the other distances oscillates between one or two order of magnitude. In the paramagnetic phase the difference is lower and moving away from the phase transition it decreases up to $|J| \approx 1.6$, when the bound for $r=\infty$ start to cross the the others. For the Ising model with $D=0.1$, the general behavior of the bounds is the same in ferromagnetic phase and for $J<-1$, but in this region the crossing value is $J \approx -1.2$. In the paramagnetic phase, for $J>1$, the difference between the bound associated to $r=\infty$ and the bounds associated to the other distances varies from one to two orders of magnitude. To generalized what we just stated, we also analyzed the effects of DM interaction up to $D=0.3$ and in this range the bounds for $J>0$ are not significantly affected by *D*. On the other hand for $J<0$, the increase *D* the makes crossing value to shift to higher values of *J*, whereas the value of the bound for $r=\infty$ at $J=-1$ decreases.

## Conclusions

In this paper, we have addressed quantum metrology in critical spin chains with anisotropy and Dzyaloshinskii–Moriya (DM) interactions, and we have shown how local and quasi-local measurements may be exploited to characterize global properties of the systems. In particular, we have shown that using local measurements collective phenomena such as different phases of the physical model can be discriminated from the analysis of a single element of the system. This implies that for systems described by the Hamiltonian ((1)), quantum correlations do not prevent the precise characterization of the system itself by measuring only one of its sub-parts.

We have also shown that upon measuring just two spins at a given distance *r*, one may exploit correlations to precisely characterize the system, i.e. to estimate the Hamiltonian parameters *J*, $\gamma$ and *D*, and that this gain persists even for infinitely distant spins, where correlations vanish. In particular, we have analytically shown that for two infinitely distant spins, the QFI and the FI for magnetization measurements coincide and are twice the corresponding single spin quantities.

For a general measurement involving two spins, the optimal distance between them depends on all the Hamiltonian parameters *J*, $\gamma$ and *D* and on the external magnetic field applied to the system. Moreover, this distance is in general different for the QFI and the FI of a magnetization measurements. In other words, the correlations among the spins may have a beneficial or a detrimental role depending on the Hamiltonian parameters and on the distance between the measured spins *r*.

We have also addressed the joint estimation of all the Hamiltonian parameters, and have shown that it is possible without intrinsic noise, regardless the distance between the measured spins (i.e. the Uhlmann matrix is vanishing $\forall r$). We have then studied the determinant of the QFI matrix in order to quantify the sloppiness of the system, and have shown that it is strongly influenced by the distance between the measured spins. Our results show that for $1 \le r \le 6$ the degree of sloppiness of the system is large, except close to the phase transitions of the system. On the other hand, in the limit of infinitely distant spins, there are wide intervals in *J* for which the sloppiness is low. To conclude the work, we have analyzed the lower bounds to precision in the multi-parameter case, finding out that the optimal distance is $r=\infty$, since the associated bound reaches the lowest values independently on the strength of the DM interaction.

## Figures and Tables

**Figure 8:**
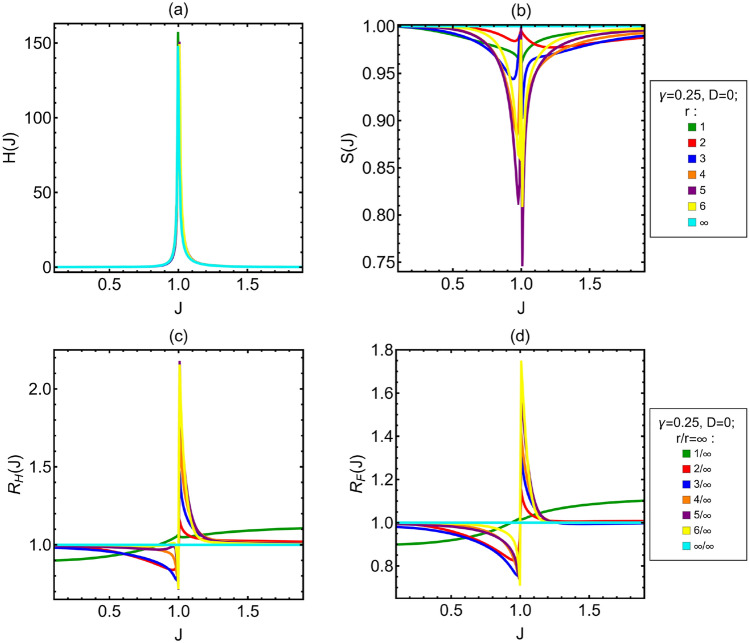
(**a**) QFI of coupling constant *J*, *H*(*J*). (**b**) Saturation associated to *J*, *S*(*J*). (**c**,**d**) Ratios between the (Q)FI of the different neighbors and QFI in the limit of infinitely distant neighbors $R_{(H)F}(J)$. All the quantities are plotted as *J* varies, $\gamma =0.25$ and $D=0$. Different curves represent different distances between the two spins measured *r*. $r=\infty$ correspond to the limit of infinitely distant spins.

**Figure 9:**
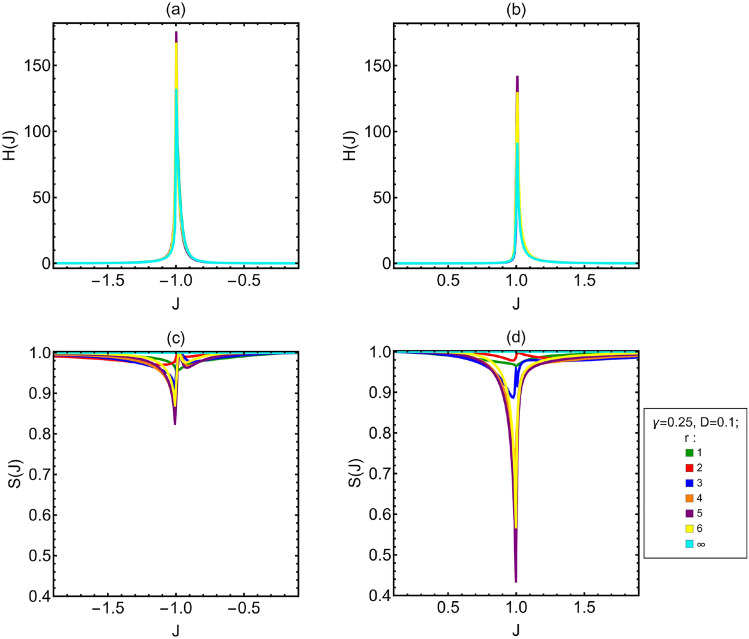
(**a**,**b**) QFI of the coupling constant *J*. (**c**,**d**) Saturation *S*(*J*) associated to the coupling constant *J*. In the limit of infinitely distant spins $H(J)=F(J)$. All the plots in figure are as *J* varies, $\gamma =0.25$ and $D=0.1$. The different curves are associated to the different values of the distance between the two spins measured *r*.

**Figure 10:**
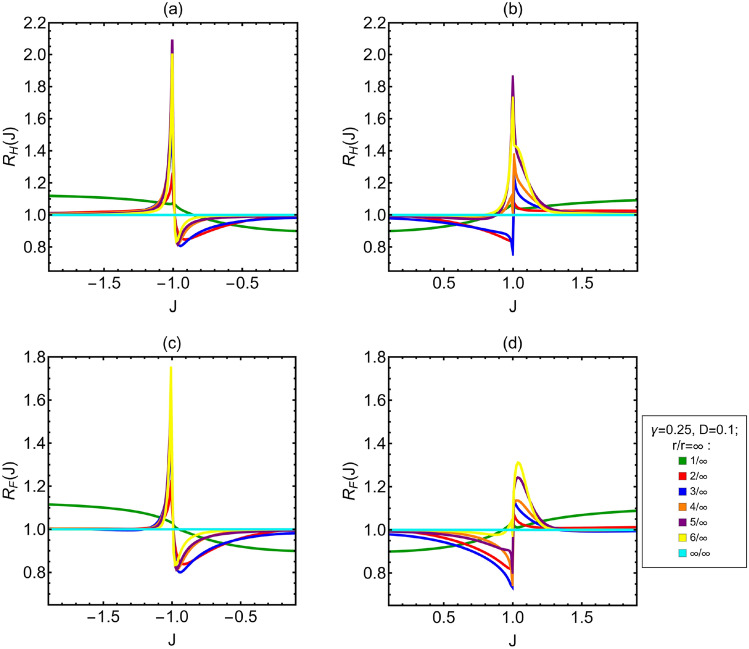
Ratios between the (Q)FI of different neighbors and (Q)FI in the limit of infinitely distant neighbors, $R_{(H)F}(J)$. All the quantities are plotted as *J* varies, $\gamma =0.25$ and $D=0.1$. Different curves represent different distances between the two spins measured *r*. $r=\infty$ correspond to the limit of infinitely distant spins.

## Data Availability

The datasets used and/or analyzed during the current study are available from the corresponding author on reasonable request.

## Appenidx A: Anisotropic XY model with $\gamma =0.25$

To support what we state in “Quasi local measurements and the role of correlations”, we provide further examples of the behavior of the (Q)FI associated to different values of $\gamma$ and *D*. We followingly report the results for the quasi-local estimation *J*, for $\gamma =0.25$ without DM interaction, $D=0$, and with DM factor $D=0.1$. The main characteristics of the (Q)FI remain the same independently on the values of $\gamma$ and *D*, promoting what we observed in “Quasi local measurements and the role of correlations” to more general considerations about our anisotropic model. A magnetization measurement is then capable to extract a consistent part of the information present in the system, for all the neighbors studied. Nevertheless, there are still differences with respect to the Ising model, since the (Q)FI assumes higher values with $\gamma =0.25$, with respect to $\gamma =1$ (see Figs. 8, 9, and 10).

## Appenidx B: Uhlmann matrix for real X-states

In this appendix, we prove that the Uhlmann matrix associated with a real X-state is always a null matrix. To do so, we start from the notions about the X-states contained in^66^. A general X-state $\rho (\lambda )$ has a matrix representation in the form

34 $$\begin{aligned} \rho (\lambda ) = \begin{pmatrix} \rho _{11} & 0 & 0 & \rho _{14} \\ 0 & \rho _{22} & \rho _{23} & 0 \\ 0 & \rho _{32} & \rho _{33} & 0 \\ \rho _{41} & 0 & 0 & \rho _{44} \end{pmatrix} . \end{aligned}$$

It can be decomposed as $\rho (\lambda ) = \rho _1(\lambda ) + \rho _2(\lambda )$. Where

35 $$\begin{aligned} \rho _1(\lambda ) = \frac{1}{2} \sum _{ \alpha = 0}^3 \ \omega _\alpha \eta _\alpha \, \ \ \ \ \ \ \ \rho _2(\lambda ) = \frac{1}{2} \sum _{ \alpha = 0}^3 \ \tilde{\omega }_\alpha \tilde{\eta }_\alpha . \end{aligned}$$

In these expressions $\eta _\alpha$ and $\tilde{\eta }_\alpha$ are defined by

36 $$\begin{aligned} &\eta _0 = |00 \rangle \langle 00| + |11 \rangle \langle 11|, \nonumber \\&\eta _1 = |00 \rangle \langle 11| + |11 \rangle \langle 00|, \nonumber \\&\eta _2 = i|11 \rangle \langle 00| - i|00 \rangle \langle 11|, \nonumber \\&\eta _3 = |00 \rangle \langle 00| - |11 \rangle \langle 11|,\nonumber \\&\tilde{\eta }_0 = |01 \rangle \langle 01| + |10 \rangle \langle 10|,\nonumber \\&\tilde{\eta }_1 = |01 \rangle \langle 10| + |10 \rangle \langle 01|, \nonumber \\&\tilde{\eta }_2 = i|10 \rangle \langle 01| - i|01 \rangle \langle 10|, \nonumber \\&\tilde{\eta }_3 = |01 \rangle \langle 01| - |10 \rangle \langle 10| \end{aligned}$$

And the $\omega _\alpha$ and $\tilde{\omega }_\alpha$ by

37 $$\begin{aligned}&\omega _0 = ( \rho _{11} + \rho _{44} ), \ \ \ \ \omega _1 = ( \rho _{14} + \rho _{41}), \nonumber\\&\omega _2 = i( \rho _{14} - \rho _{41} ), \ \ \ \ \omega _3 = ( \rho _{11} - \rho _{44} ), \nonumber\\&\tilde{\omega }_0 = ( \rho _{22} + \rho _{33} ), \ \ \ \ \tilde{\omega }_1 = ( \rho _{23} + \rho _{32}), \nonumber \\&\tilde{\omega }_2 = i( \rho _{23} - \rho _{32} ), \ \ \ \ \tilde{\omega }_3 = ( \rho _{22} - \rho _{33} ). \end{aligned}$$

If $\rho (\lambda )$ is real (as in our case), we have $\rho _{ij}=\rho _{ji}^{*}=\rho _{ji}$, so

38 $$\begin{aligned} &\omega _1 = 2 \rho _{14}=2b_{-}, \ \ \ \ \omega _2 = 0, \nonumber \\&\tilde{\omega }_1 = 2 \rho _{23}=2b_{+}, \ \ \ \ \tilde{\omega }_2 = 0. \end{aligned}$$

Also the Symmetric Logarithmic Derivative associated to $\rho (\lambda )$ can be decomposed as $\mathscr {L}_{\lambda } = \mathscr {L}_{\lambda }^{(1)}+ \mathscr {L}_{\lambda }^{(2)}$, where $\mathscr {L}_{\lambda }^{(1)}$ is the SLD associated to $\rho _1(\lambda )$ and $\mathscr {L}_{\lambda }^{(2)}$ the SLD associated to $\rho _2(\lambda )$. $\mathscr {L}_{\lambda }^{(1)}$ and $\mathscr {L}_{\lambda }^{(2)}$ can be written as

39 $$\begin{aligned}&\mathscr {L}_{\lambda }^{(1)} =\sum _{ \alpha = 0}^3 \ f_\alpha \eta _\alpha \, \nonumber \\&\mathscr {L}_{\lambda }^{(2)} =\sum _{ \alpha = 0}^3 \ \tilde{f}_\alpha \tilde{\eta }_\alpha . \end{aligned}$$

where

40 $$\begin{aligned} &f_0 = \frac{\omega _0(\partial _\lambda \omega _0)- \sum _i \omega _i (\partial _\lambda \omega _i)}{\omega _0^2 - \sum _i \omega _i^2}, \nonumber\\&f_i = \frac{\partial _\lambda \omega _i - f_0 \omega _i}{\omega _0}, \nonumber\\&\tilde{f}_0 = \frac{\tilde{\omega }_0(\partial _\lambda \tilde{\omega }_0)- \sum _i \tilde{\omega }_i (\partial _\lambda \tilde{\omega }_i)}{\tilde{\omega }_0^2 - \sum _i \tilde{\omega }_i^2}, \nonumber\\&\tilde{f}_i = \frac{\partial _\lambda \tilde{\omega }_i - \tilde{b}_0 \tilde{\omega }_i}{\tilde{\omega }_0}, \ \ i=1,2,3. \end{aligned}$$

When $\rho (\lambda )$ is real we have $f_2=\tilde{f}_2=0$, since $\omega _2=\tilde{\omega }_2=0$, and therefore the SLD $\mathscr {L}(\lambda ) = \mathscr {L}_{\lambda }^{(1)} + \mathscr {L}_{\lambda }^{(2)}$ is symmetric and has a X structure. This is the main point for the following. Up to now we used the formalism of the single parameter estimation for simplicity ($\lambda$ is scalar). From now on we use the formalism of the multi-parameter estimation, so now $\varvec{\lambda }$ is the vector of the parameters to be estimated. Now let’s look for the Uhlmann matrix (Eq.(26)). When $\rho (\lambda )$ is real and has an X structure, we can write the SLD associate to a generic parameter $\mu \in \varvec{\lambda }$ and the SLD associate to a generic parameter $\nu \in \varvec{\lambda }$ as

41 $$\begin{aligned} & \mathscr {L}_{\mu } =\begin{pmatrix} \mu _{11} & 0 & 0 & \mu _{14} \\ 0 & \mu _{22} & \mu _{23} & 0 \\ 0 & \mu _{23} & \mu _{33} & 0 \\ \mu _{14} & 0 & 0 & \mu _{44} \end{pmatrix}, \nonumber \\ & \mathscr {L}_{\nu } = \begin{pmatrix} \nu _{11} & 0 & 0 & \nu _{14} \\ 0 & \nu _{22} & \nu _{23} & 0 \\ 0 & \nu _{23} & \nu _{33} & 0 \\ \nu _{14} & 0 & 0 & \nu _{44} \end{pmatrix}. \end{aligned}$$

Now to find the elements of the Uhlmann matrix we can just apply

42 $$\begin{aligned} {\varvec{U}}_{\mu \nu }\varvec{(\lambda )} = Tr\left[ \rho _{\varvec{\lambda }}\frac{\mathscr {L}_{\mu }\mathscr {L}_{\nu }-\mathscr {L}_{\nu }\mathscr {L}_{\mu }}{2}\right] = \hbox {Tr}\left[ M\right] . \end{aligned}$$

Performing the algebra we find the diagonal elements $M_{ii}$ of the matrix inside the trace in the equation above (Eq.(42)). These are

43 $$\begin{aligned}&M_{11}=\frac{1}{2}((-\mu _{11}+\mu {44})\nu _{14}+\mu _{14}(\nu _{11}-\nu _{44}))b_{-}, \\&M_{22}=\frac{1}{2}((-\mu _{22}+\mu {33})\nu _{23}+\mu _{23}(\nu _{22}-\nu _{33}))b_{+}, \\&M_{33}=\frac{1}{2}((\mu _{22}-\mu {33})\nu _{23}+\mu _{23}(-\nu _{22}+\nu _{33}))b_{+}, \\&M_{44}=\frac{1}{2}((\mu _{11}-\mu {44})\nu _{14}+\mu _{14}(-\nu _{11}+\nu _{44}))b_{-}, \end{aligned}$$

so $M_{11}=-M_{44}$ and $M_{22}=-M_{33}$. For this reason taking trace of *M* we obtain

44 $$\begin{aligned} {\varvec{U}}_{\mu \nu }\varvec{(\lambda )} = 
0, \ \ \ \ \forall \ \mu ,\nu \in \varvec{\lambda }. \end{aligned}$$

This means that for the systems described by a real X-state the Uhlmann matrix is always null and the Symmetric Logarithmic Derivatives commutes weakly among each other. For this reason all the systems of this kind are classified as *asymptotically classical systems*^67^.
